# Birth and Health Outcomes of Children Migrating With Parents: A Systematic Review and Meta-Analysis

**DOI:** 10.3389/fped.2022.810150

**Published:** 2022-07-13

**Authors:** Ruixia Chang, Chunan Li, Haiqin Qi, Ya Zhang, Jianduan Zhang

**Affiliations:** Department of Maternal and Child Health, School of Public Health, Tongji Medical College, Huazhong University of Science and Technology, Wuhan, China

**Keywords:** migrant children, health outcomes, meta-analysis, healthy migration effect, birth outcomes

## Abstract

**Objective:**

To examine the birth and health outcomes of children migrating with parents internationally and domestically, and to identify whether the healthy migration effect exist in migrant children.

**Methods:**

Five electronic databases were searched for cross-sectional, case-control, or cohort studies published from January 1, 2000 to January 30, 2021and written by English language, reporting the risk of health outcomes of migrant children (e.g., birth outcome, nutrition, physical health, mental health, death, and substance use) We excluded studies in which participants' age more than 18 years, or participants were forced migration due to armed conflict or disasters, or when the comparators were not native-born residents. Pooled odd ratio (OR) was calculated using random-effects models.

**Results:**

Our research identified 10,404 records, of which 98 studies were retrained for analysis. The majority of the included studies (89, 91%) focused on international migration and 9 (9%) on migration within country. Compared with native children, migrant children had increased risks of malnutrition [OR 1.26 (95% CI 1.11–1.44)], poor physical health [OR 1.34 (95% CI 1.11–1.61)], mental disorder [OR 1.24 (95% CI 1.00–1.52)], and death [OR 1.11 (95% CI 1.01–1.21)], while had a lower risk of adverse birth outcome [OR 0.92 (95% CI 0.87–0.97)]. The difference of substance use risk was not found between the two groups.

**Conclusion:**

Migrant children had increased risk of adverse health outcomes. No obvious evidence was observed regarding healthy migration effect among migrant children. Actions are required to address the health inequity among these populations.

**Systematic Review Registration:**

https://www.crd.york.ac.uk/prospero/#myprospero, identifier: CRD42021214115.

## Introduction

Migration is a global phenomenon with nearly one in seven individuals being a migrant (1). The majority are labor migrants who relocate to more developed areas, seeking employment opportunities, either internationally or domestically. Others are forced migrants because of wars, conflicts, or natural disasters. A growing number of children are compelled to migrate with their parents. According to the International Organization for Migration, the number of children migrating with their families beyond a country's border reached 37.9 million in 2019 (1). Similarly, the number of children migrating within a country (e.g., from rural to urban) is also spiking. In China, about 20.8% of the migrant population in 2010 were children younger than 14 years old (2).

Migrants are usually at a relatively lower socioeconomic ladder and have less access to public welfare, such as healthcare services and education (3). However, migrant adults present similar or better health outcomes compared to native populations in multiple health indices, including pregnancy outcomes, self-reported health, and adult mortality (4, 5). This phenomenon, known as the “migrant paradox” or “healthy migrant effect,” has been much debated (6). Evidence regarding the impacts of migration on migrant children's health status is inconsistent. Some migrant children experienced overall better health outcomes than the native-born children. In Portugal, children who migrated from other counties have a lower risk of being low birth weight (LBW) and small for gestational age (SGA) than native children (5). In contrast, the migratory process can generate unfavorable social and medical care conditions, placing the health of migrant children at risk. International migrant children in European and American countries have worse physical health (7), more mental health problems (8), and increased risks of fetal and infant mortality (9). These inconsistent observations may be related to population origins, migration types, and health indices used in the studies (10–12). Therefore, it is important to systematically examine the health status of migrant children and to understand the extent the health of these children is affected by migration and how the impacts may vary in regard to various health outcomes at birth and in later life.

No comprehensive assessment is available regarding the health status of migrant children across all the key areas of health. To address this study gap, we performed a systematic review and meta-analysis to evaluate the impact of migration on major health indicators, including children's birth outcome, nutrition, physical health, mental health, death, and substance use. We also examined whether the migration type (international or internal) differentially influences the health of these children. This is in response to the debate regarding the migrant paradox among children populations.

## Methods

### Search Strategy and Selection Criteria

For this systematic review and meta-analysis, we searched five electronic databases, including PubMed, Embase, Web of Science, Cochrane, and Scopus from January 1, 2000 to January 30, 2021. The full search strategy is provided in the Appendix. Based on literature review, we decided to investigate six categories of health outcomes: birth outcomes, nutrition, physical health, mental health, death, and substance use. We searched observational studies (e.g., cohort, case-control, or cross-section) reporting the risk of health outcomes that included migrant children aged 0–18 years. Both internal and international migrations were included. We defined international migrant children as those with at least one foreign-born parent, irrespective of the child's birth place, including first-generation and second-generation immigrants (13). Internal migrant children refer to children who have lived in the host city for more than 6 months while holding a non-local household residency, such as rural-to-urban migration (14). The comparator group consisted of native-born children (e.g., children and both parents without migration background) (15). We excluded the studies on refugee children who migrated due to armed conflict, disasters, or political, religious or ethnic persecution. Those with a comparator group of non-natives were also excluded. The initial literature search and screening to assess eligibility was done by two reviewers (HQ and Y). Any discrepancies about study inclusion were resolved through discussion with RX. Data were extracted by two reviewers (RX and CN) and checked by two others (HQ and Y). Studies that reported results as odds ratios (ORs) or included data that enable the calculation of ORs were retained for analysis. This study is reported in accordance with the PRISMA guidelines (16) (Appendix).

We summarized the health outcomes from all the included studies as follows. Birth outcomes included low birth weight (LBW), high birth weight (HBW), and preterm birth. Nutritional outcomes were overweight/obesity, underweight, and iron deficiency anemia. Physical health included oral, gastrointestinal, respiratory, allergic, and congenital diseases. Mental health covered depression, attention deficit hyperactivity disorder (ADHD), autistic spectrum disorder (ASD), schizophrenia, suicide attempt. Deaths referred to fetal, perinatal, neonatal, post-neonatal, and infant deaths. Substance use included tobacco, alcohol, and cannabis (Appendix).

### Data Analysis

The quality assessment for all included studies was done independently by two reviewers (RX and HQ) using an adapted version of the Newcastle Ottawa Scale (Appendix). Studies with a high or unclear risk of bias across five or more domains were assessed as having high risk of bias. For each article ultimately included, we extracted data on the name of authors, publishing year, study country, study design, age of participants, sample size, and health outcomes using self-designed data extraction sheets. We also extracted ORs or recalculated pertinent ORs using available data.

We estimated pooled OR with 95% confidence intervals (CIs) for the risk of health outcomes using a random-effects model. The *I*^2^ statistic was used to estimate the proportion of total variation among the pooled studies due to heterogeneity. We performed subgroup analyses of study region (e.g., Europe vs. non-Europe) to assess the source of heterogeneity. Subgroup analyses were also conducted per migration type if possible, and the risk of each health outcome was assessed by host countries (the countries with at least two studies in each selected health outcome). We explored the potential risk of publication bias using Begg's and Egger's tests. We used forest plots to show the OR and 95% CIs for each study and the pooled estimates. A sensitivity analysis was performed to assess the robustness of our conclusions by excluding studies with quality score less than five. We used meta-regression to assess the effect of sample size (continuous), study design (cross-section vs. non- cross-section), publish year (<2010 vs. ≥2010), and participant's age (continuous) on health outcomes. All statistical analyses were done using Stata (version 12.0). The study was registered with PROSPERO (number: CRD42021214115).

## Results

### Characteristics of the Included Studies

**Figure 1:**
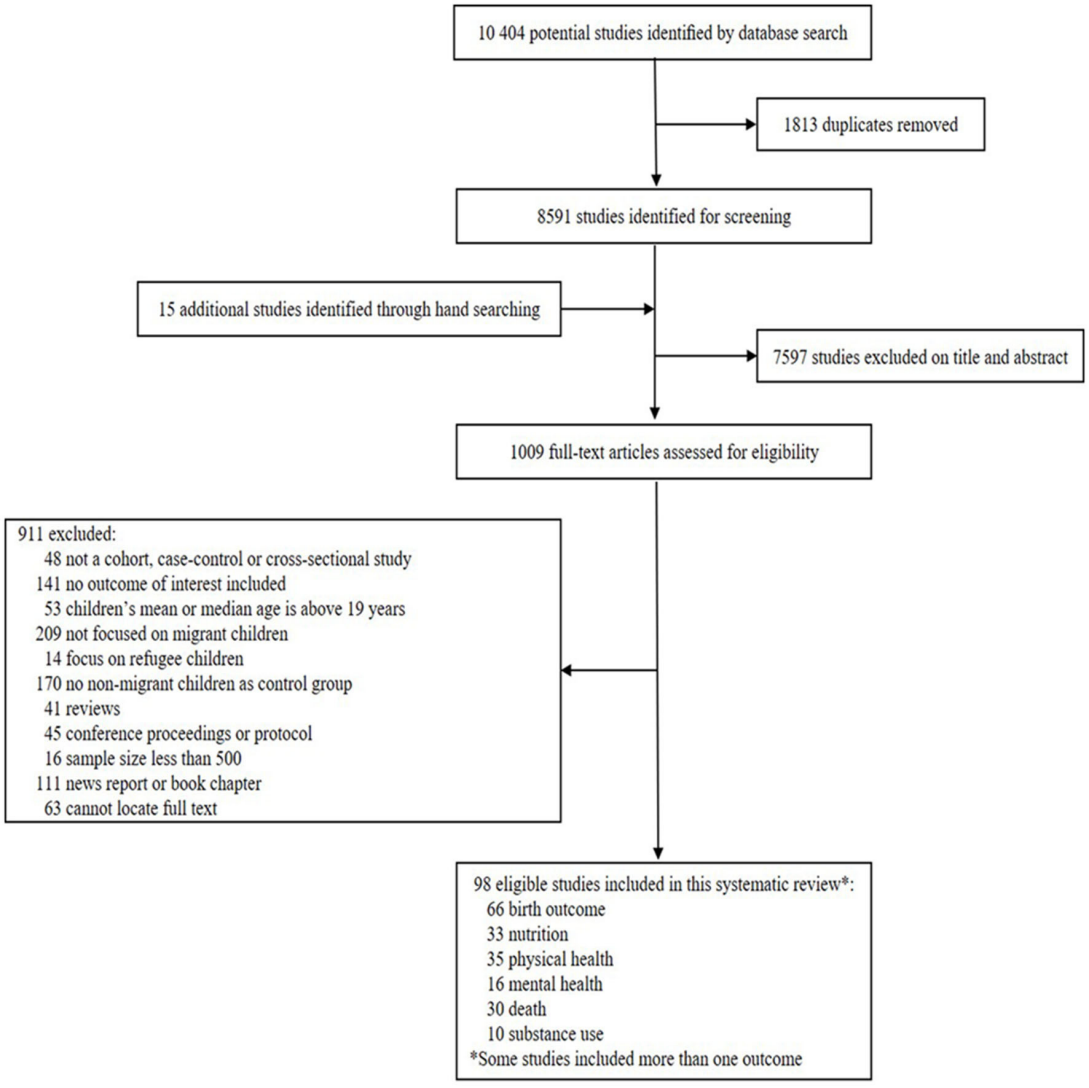
PRISMA flow diagram. *Some studies included more than one outcome.

Among 10,404 references identified through the literature search, full-text copies of 1,009 articles were retrieved and screened, with 98 articles selected for analysis. The PRISMA flow diagram and study characteristics were shown in Figure 1 and Table 1. Among the 98 included articles, 90.8% (89) involved international migration, 66.3% (65) studies were conducted in European countries, and 75.5% (74) were cross-sectional studies. Overall, 79.6% (78) of studies included children <10 years of age. The quality of the included studies varied, with 24.5% (24) studies bearing high or unclear risk of bias (Appendix). Birth outcomes were most commonly examined (*n* = 55), followed by physical health status (*n* = 34), nutrition status (*n* = 29), death (*n* = 28), mental health status (*n* = 16), and substance use (*n* = 8) (Appendix).

**Table 1:** Characteristics of the included studies (*N* = 98).

**Author [reference]**	**Year**	**Country**	**Design**	**Age**	**No. of migrants**	**No. of controls**	**Health outcome**	**Measurement/Instrument**
Kana et al. (5)	2019	Portugal	CS	—	386	8,171	Birth outcome (LBW, Preterm birth)	LBW: birth weight <2,500 g Preterm birth: delivered at <37 completed weeks
Gillet et al. (9)	2014	Belgium	CS	—	129,200	261,566	Birth outcome (LBW, Preterm birth) Death (Neonatal death, Post-neonatal death, Infant death)	LBW: birth weight <2,500 g Preterm birth: Gestational age (week) <37 Infant death was defined as the sum of early neonatal (death at 0–6 days), late neonatal (7–27 days), and post-neonatal (28–364 days) deaths.
Cebolla-Boado and Salazar (17)	2016	Spain	CS	—	71,758	287,153	Birth outcome (LBW, HBW)	LBW: birth weight ≤ 2,500 g HBW: birth weight >4,000 g
Forna et al. (18)	2003	US	Cohort	—	13,465	36,439	Birth outcome (LBW, Preterm birth)	LBW: birth weight <2,500 g Preterm birth: delivery before 37 weeks' gestation
Sandra et al. (19)	2015	Spain	CS	—	72,567	599,660	Birth outcome (LBW, HBW)	LBW: birth weight <2,500 g HBW: birth weight >4,000 g
Besharat et al. (20)	2014	Sweden	Cohort	—	336	2,181	Birth outcome (LBW, HBW, Preterm)	LBW: birth weight <2,500 g HBW: birth weight (gram) ≥ 4,000 g Preterm birth: delivered at <37 completed weeks
Racape et al. (21)	2016	Belgium	CS	—	334,150	1,029,471	Birth outcome (LBW) Death (Perinatal death)	LBW: birth weight <2,500 g Perinatal death: fetal deaths from 22 weeks of gestation until 7 days after birth
Lehti et al. (22)	2013	Finland	CC	—	347	5,300	Birth outcome (LBW, Preterm) Mental health (ASD)	LBW: birth weight <2,500 g Preterm birth: Gestational age <37 weeks ASD: clinical assessment ICD-9/ICD-10
Milewski and Peters (23)	2014	Germany	Cohort	—	427	1,214	Birth outcome (LBW, HBW)	LBW: birth weight <2,500 g HBW:birth weight ≥4,000 g
Ratnasiri et al. (24)	2020	US	CS	—	446,724	611,253	Birth outcome (LBW, Preterm birth) Death (Neonatal death, Post-neonatal death)	LBW: birth weight <2,500 g Preterm birth: Gestational age (week) <38 Neonatal death: death before 28 days of age Post-neonatal death: death at 28d-1year
Hessol and Fuentes-Afflick (25)	2014	US	CS	—	410,284	231,190	Birth outcome (LBW, HBW, Preterm birth)	LBW: birth weight <2,500 g HBW: birth weight ≥4,000 g Preterm birth: <37 completed weeks' gestation
Castello et al. (26)	2012	Spain	CS	—	5,926	15,782	Birth outcome (LBW, Preterm birth)	LBW: birth weight <2,500 g Preterm birth: Gestational age <37 weeks
Juarez and Revuelta-Eugercios (27)	2014	Spain	CS	—	323,856	1,061,924	Birth outcome (LBW, HBW, Preterm birth)	LBW: birth weight <2,500 g HBW: macrosomia >4,500 g Preterm birth: <37 completed gestational weeks
Fuster et al. (28)	2014	Spain	CS	—	412,906	1,874,913	Birth outcome (LBW, preterm) Death (fetal death)	LBW: birth weight <2,500 g Preterm birth: gestational age <38 week Fetal death: the number of late fetal deaths per 1,000 deliveries (both live births and fetal deaths)
Racape et al. (29)	2010	Belgium	Cohort	—	56,061	74,767	Birth outcome (LBW, Preterm birth) Death (Fetal mortality, Neonatal mortality, Post-neonatal mortality)	LBW: birth weight <2,500 g Preterm birth: Gestational age <37weeks Fetal mortality: fetal deaths of 22 or more weeks of gestation; Neonatal mortality: death at 0–27 days of life per 1,000 live births; Post-neonatal mortality: death at 28–364 days of life per 1,000 live births
Glick et al. (30)	2009	US	Cohort	—	2,300	7,300	Birth outcome (LBW)	LBW: birth weight <2,500 g
Farre (31)	2013	Spain	CS	—	233,518	1,773,102	Birth outcome (LBW, Preterm birth) Death (Perinatal death)	LBW: birth weight <2,500 g Preterm birth: Gestational age (week) <38 Perinatal death: death within 24 h after birth
Nancy et al. (32)	2015	US	CS	—	305	1,361	Birth outcome (LBW)	LBW: Birth weight <2,500 g
Lehti et al. (33)	2016	Finland	CC	—	1,730	47,803	Birth outcome (LBW, Preterm) Mental health (ADHD)	LBW: birth weight <2,500 g Preterm birth: gestational age <37 weeks ADHD: clinical assessment ICD-9/ICD-10
Gissler et al. (34)	2003	Sweden	CS	—	34,357	110,008	Birth outcome (LBW) Death (Fetal death, Neonatal death, Perinatal death)	LBW: birth weight <2,500 g Death at the age of 7 days (stillbirth, early neonatal death or living at the end of the perinatal period).
Madan et al. (35)	2006	US	CS	—	2,418,501	4,005,671	Birth outcome (LBW) Death (Neonatal death, Post-neonatal death)	LBW: birth weight <2,500 g Neonatal deaths: death before 28 days of age per 1,000 live births) Post-neonatal deaths: 28 days to 1 year
Auger et al. (36)	2008	Canada	CS	—	43,396	54,954	Birth outcome (LBW, Preterm birth)	LBW: birth weight <2,500 g Preterm birth: delivery at <37 completed weeks of gestation
Bastola et al. (37)	2020	Finland	CS	—	31,454	350,548	Birth outcome (LBW, HBW, Preterm birth) Death (Post-neonatal death, Neonatal death, Fetal death)	LBW: birth weight <2,500 g HBW: birth weight≥4,000 g Preterm birth: ≤ 36 week + 6 days Post-neonatal death: 28 days to 1 year of death Neonatal mortality: death of a live-born child within the first 28 days of life Fetal death: Stillbirths
Marcon et al. (38)	2011	Italy	CS	3–14 years	641	2,980	Birth outcome (LBW, HBW) Physical health (Pneumonia, Eczema)	LBW: birth weight <2,500 g HBW: birth weight >4,200 g Pneumonia and Eczema: Self-reported symptoms
Besharat Pour et al. (39)	2017	Sweden	Cohort	—	299	1,979	Birth outcome (HBW, Preterm birth)	HBW: Birth weight≥4,000 g Preterm birth: Gestational age <38 weeks
Reeske et al. (40)	2013	Germany	Cohort	—	384	903	Birth outcome (HBW)	HBW: Birth weight≥4,000 g
Choi et al. (41)	2019	Australia	Cohort	—	601,299	1,735,724	Death (Neonatal death, Fetal death) Birth outcome (Preterm birth)	Death <28 days among livebirths Preterm birth: Gestational age <37 weeks
Essen et al. (42)	2000	Sweden	CS	—	5,211	10,784	Birth outcome (Preterm birth) Death (Perinatal death)	Preterm birth: Gestational age <37 weeks Perinatal death: stillbirth (fetus >28 weeks of gestation) and death within the first week of life
Vik et al. (43)	2019	Norway	CS	—	198,520	115,6444	Death (fetal death) Birth outcome (Preterm birth)	Stillbirth: a pregnancy loss at ≥22weeks of gestation or with a birthweight ≥500 g if data on gestational age were missing Moderately preterm: gestational age 28–36 weeks
Anil Kumar (44)	2016	India	CS	0–4 years	11,327	2,488	Nutrition (Overweight/ obesity, Anemia) Physical health (Diarrhea)	Overweight/obesity: BMI scores Anemia: blood hemoglobin level Diarrhea: self-reported
Liu et al. (45)	2016	China	CS	5–12 years	3,057	6,860	Nutrition (Overweight/ Obesity)	World Health Organization reference 2007 Overweight: +1 SD < BMI-for-age z-score ≤ +2 SD); Obesity: BMI-for-age z-score > +2 SD
Ji et al. (14)	2016	China	CS	10.7 ± 0.94 years	991	650	Nutrition (Overweight/ Obesity) Physical health (Caries experience, Diarrhea)	Overweight/obesity: based on cutoff of the Working Group on Obesity of China Caries: referred to those in both deciduous and permanent teeth Diarrhea: Parents reported symptom
Lin et al. (46)	2011	Taiwan	CS	7–12 years	157	519	Nutrition (Overweight) Mental health (Depression)	Overweight: BMI≥85th percentile based on Department of Health criteria, Taiwan, ROC. Depression: The “Depression Screen Scale for Children and Adolescents” developed by Kao-Pin Chang
De Carli et al. (47)	2018	Italy	CS	11–12 years	353	847	Nutrition (Overweight/obesity, Underweight)	The International Obesity Task Force cut-offs
Zulfiqar et al. (48)	2018	Australia	Cohort	2–11 years	1,799	2,434	Nutrition (Overweight/obesity)	The International Obesity Task Force standard
Maximova et al. (13)	2011	Canada	CS	11.2 ± 1.1 years	5,261	1,131	Nutrition (Overweight/obesity)	The International Obesity Task Force standard
Lindstrom et al. (49)	2014	Sweden	CS	15–16 years	2,423	7,195	Nutrition (Overweight/obesity) Physical health (Asthma) Substance use (Tobacco use)	Boy: overweight/obesity 23.29–28.29/28.30; girl: 23.94–29.10/29.11
Esteban-Gonzalo et al. (50)	2014	Spain	CS	13–17 years	335	1,742	Nutrition (Overweight/ obesity) Mental health (Depression) Substance use (Tobacco use)	Overweight/obesity: using the BMI age- and gender-specific cut-offs proposed by Cole et al.) 27, Depression: self-reported medical diagnosis of depression Tobacco use: self-reported questionnaire
Besharat Pour et al. (51)	2014	Sweden	Cohort	8 years	561	2,028	Nutrition (Overweight/obesity)	Age- and sex-adjusted BMI corresponding to adult BMI ≥ 25 kg/m^2^
Furthner et al. (52)	2017	Austria	CS	13.8 years	827	2,103	Nutrition (Overweight/Obesity)	Overweight: 85th−95th BMI percentile Obesity: ≥95th BMI percentile
Burgi et al. (53)	2010	Switzerland	CS	5.1 ± 0.60 years	391	151	Nutrition (Overweight/obesity)	International Obesity Task Force (IOTF)
Iguacel et al. (54)	2018	Spain	Cohort	2.0–9.9 years	1,156	7,427	Nutrition (Overweight/Obesity)	extended International Obesity Task Force criteria
Khanolkar et al. (55)	2013	Sweden	CS	4–5 years	1,286	9,342	Nutrition (Overweight/Obesity)	International Obesity Task Force
Thi et al. (56)	2019	Germany	CS	5–7 years	1,080	2,623	Nutrition (Overweight/Obesity)	BMI is above the 90th percentile according to the BMI reference systems of Kromeyer-Hauschild
Will et al. (57)	2005	Germany	CS	6–7 years	258	265	Nutrition (Overweight/Obesity)	The International Obesity Task Force, using international reference values based on data from six countries
Zhou et al. (58)	2018	Germany	CS	—	19,245	31,441	Nutrition (Overweight/obesity)	BMI≥90th percentile based on the German national reference
Méroc et al. (15)	2019	Belgium	CS	10–11 years	2,319	553	Nutrition (Overweight/obesity)	The International Obesity Task Force standard
Labree et al. (59)	2015	Finland	CS	8–9 years	397	1,546	Nutrition (Overweight/Obesity, Underweight)	The International Obesity Task Force standard
**Author [reference]**	**Year**	**Country**	**Design**	**Age**	**No. of migrants**	**No. of controls**	**Health outcome**	**Measurement/Instrument**
Brettschneider et al. (60)	2011	Germany	CS	11–17 years	518	2,949	Nutrition (Overweight)	BMI >90th percentile based on the national German reference
Vorwieger et al. (61)	2018	Germany	CS	7.57 ± 0.42 years	245	508	Nutrition (Abdominal obesity)	WHtR ≥0.5
Nagel et al. (62)	2009	Germany	CS	7.6 ± 0.4 years	317	762	Nutrition (Overweight/obesity)	The International Obesity Task Force standard
Beyerlein et al. (63)	2014	Germany	CS	3–17 years	474	8,507	Nutrition (Overweight)	International Obesity Task Force (IOTF)
Prusty and Keshri (64)	2015	India	CS	0–59 months	13,220	5,617	Nutrition (Underweight)	WFA-Z < -2SD
Saunders et al. (65)	2016	Canada	CS	12–72 months	1,244	1,268	Nutrition (Anemia)	Hemoglobin level <110 g/L (WHO recommendation)
Hu et al. (66)	2014	China	CS	6–23 months	667	321	Nutrition (Anemia)	Hemoglobin level <110 g/L (WHO recommendation)
Julihn et al. (67)	2010	Sweden	Cohort	13–19 years	5,134	10,404	Physical health (Caries experience)	Clinical examination of DMFT
Christensen et al. (68)	2010	Denmark	CS	5–15 years	3,571	9,058	Physical health (Caries experience)	Clinical examination of DMFT
van Meijeren et al. (69)	2019	Netherlands	Cohort	9 years	611	2,510	Physical health (Caries experience)	Clinical examination of DMFT
Ferrazzano et al. (70)	2019	Italy	CS	12–14 years	183	370	Physical health (Caries experience)	Clinical examination of DMFT
van der Tas et al. (71)	2016	Netherlands	CS	4.96 years	1,403	2,957	Physical health (Caries experience)	Clinical examination of DMFT
Almerich-Silla and Montiel-Company (72)	2007	Spain	CS	12–15 years	54	825	Physical health (Caries experience)	Clinical examination of DMFT
Bissar et al. (73)	2014	Germany	CS	4.1 ± 0.8 years	265	698	Physical health (Caries experience)	Clinical examination of DMFT
Baggio et al. (74)	2015	Switzerland	CS	36–71 months	398	457	Physical health (Caries experience)	Clinical examination of DMFT
Bardin et al. (75)	2019	Italy	Cohort	0–14 years	21,817	191,345	Physical health (Gastroenteritis, Pneumonia, Asthma)	ICD-9
Charania et al. (76)	2020	New Zealand	CS	0–5 years	125,511	567,408	Physical health (Gastroenteritis, Pneumonia)	Hospitalization event
Li et al. (77)	2019	China	CS	12–15 years	3,477	2,213	Physical health (Pneumonia, Asthma, Eczema)	Physician-diagnosed
Migliore et al. (78)	2007	Italy	CS	6–7/13–14 years	1,012	28,293	Physical health (Pneumonia, Asthma)	Self-reported questionnaire
Keet et al. (79)	2012	US	CS	11.4 years	341	3,209	Physical health (Asthma, Eczema, Food allergy)	Physician-diagnosed
Svendsen et al. (80)	2009	US	CS	9–11 years	2,026	4,370	Physical health (Asthma, Food allergy)	Physician-diagnosed
Radhakrishnan et al. (81)	2019	Canada	Cohort	0–18 years	422,305	968,256	Physical health (Asthma)	ICES (institute for clinical Evaluation Sciences) database
Apfelbacher et al. (82)	2011	Germany	CS	0–17 years	2,550	14,640	Physical health (Eczema)	Physician-diagnosed
Koplin et al. (83)	2014	Australia	Cohort	11–15 months	535	3,023	Physical health (Food allergy)	Food allergy to egg, peanut or sesame was defined as a positive oral food challenge in sensitized infants (SPT wheal ≥ 2 mm or specific IgE ≥ 0.35 ku/l)
Ramadhani et al. (84)	2009	US	CC	—	575	539	Physical health (Heart defects, Neural tube defect)	Surveillance registries system
Kang et al. (85)	2016	China	CS	6–13 years	325,940	214,634	Physical health (Heart defects)	Clinical cardiovascular examination
Velie et al. (86)	2006	US	CC	—	265	606	Physical health (Neural tube defect)	California Birth Defects Monitoring system
Kim et al. (87)	2018	US	CS	8–18 years	1,013	1,361	Mental health (Depression)	MFQ (Mood and Feelings Questionnaire)
Fuhrmann et al. (88)	2014	US	CS	6.2 ± 0.4 years	118	535	Mental health (Depression)	PFC (Preschool Feelings Checklist)
Adriaanse et al. (89)	2014	Netherlands	CS	12.9 ± 1.8 years	576	702	Mental health	The Strengths and Difficulties Questionnaire (SDQ)
Wang et al. (90)	2017	China	CS	11.04 ± 0.04 years	731	451	Mental health	SDQ
van der Ven et al. (91)	2013	Netherlands	Cohort		26,599	80,354	Mental health (ASD)	DSM-IV diagnosis
Wandell et al. (92)	2020	Sweden	Cohort	<18 years	1,149,504	2,873,645	Mental health (ASD)	DSM-IV diagnosis
Magnusson et al. (93)	2012	Sweden	CC	0–17 years	9,396	34,567	Mental health (ASD)	DSM-IV diagnosis
Weiser et al. (94)	2008	Israel	CS	7.7 ± 3.7 years	639,203	22,589	Mental health (Schizophrenia)	ICD-9 and ICD-10
Hjern et al. (95)	2004	Sweden	CS	—	87,988	1,056,225	Mental health (Schizophrenia)	ICD-9/ICD-10
Pedersen et al. (96)	2012	Denmark	Cohort	—	202	1,639	Mental health (Schizophrenia)	ICD-10
Lu et al. (97)	2020	China	CS	13.67 ± 1.52 years	1,858	2,359	Mental health (Suicide attempt)	Self-injurious thoughts and behaviors (SITBs)
Vazsonyi et al. (98)	2017	Switzerland	CS	17.85 ± 1.21years	741	6,546	Mental health (Suicide attempts)	Self-reported questionnaire
Villadsen et al. (99)	2010	Northern Europe	CS	—	265,135	9,649,736	Death (Neonatal mortality, Fetal death)	Death within 0–27 days of birth
Barona-Vilar et al. (100)	2014	Spain	CS	—	40,834	162,043	Death (Perinatal death)	The number of fetal and neonatal deaths per 1,000 total births
Vang (101)	2016	Canada	CS	—	514,247	2,856,394	Death (Post-neonatal death, Neonatal death)	Neonatal death: 0 to 27 days and Post-neonatal death: 28 to 364 days
Rosenberg et al. (102)	2002	US	CS	—	72,293	130,681	Death (Infant death)	Death before first birthday
Landale et al. (103)	2006	US	CS	—	4,342	715	Death (Infant death)	Died before reaching the age of 1 year
Troe et al. (104)	2007	Netherlands	CS	—	30,331	3,838	Death (Infant death)	Died before reaching the age of 1 year
Abebe et al. (105)	2015	Norway	CS	14–17 years	2,932	8,002	Substance use (Cannabis use, Tobacco use, Alcohol use)	Self-report questionnaire
Slonim-Nevo et al. (106)	2006	Russia	CS	15–17 years	396	127	Substance use (Tobacco use, Alcohol use)	Self-report questionnaire
Donath et al. (107)	2016	Germany	CS	14.88 ± 0.74 years	2,277	7,235	Substance use (Cannabis use)	Self-report questionnaire

*CS, cross-sectional; CC, case-control; LBW, low birthweight; HBW, high birthweight; ADHD, attention deficit hyperactivity disorder; ASD, autistic spectrum disorder. — represents the data cannot be obtained*

### Birth Outcomes

**Figure 2:**
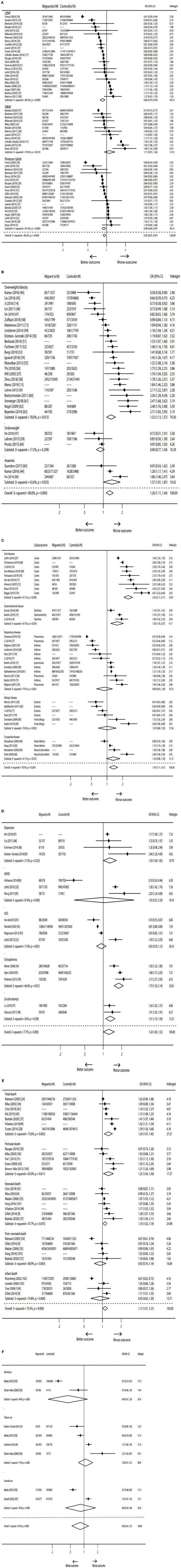
Forest plot of ORs for health outcomes. **(A)** Birth outcome. **(B)** Nutrition. **(C)** Physical health. **(D)** Mental health. **(E)** Death. **(F)** Substance use.

Migrant children had a lower risk of adverse birth outcome [OR 0.92 (95% CI 0.87–0.97)] than non-migrant children, including lower risk of LBW [OR 0.86 (95% CI 0.79–0.94)] and preterm birth [OR 0.90 (95% CI 0.84–0.97)] (Figure 2A). Although high statistical heterogeneity across birth outcomes was observed, it was reduced after subgroup and sensitivity analysis. In the subgroup analyses by region (Table 2), although no significant difference of overall adverse birth outcomes was found between migrant children and native ones in European countries [OR 0.95 (95% CI 0.90–1.02)], a lower risk of low birthweight was identified. In non-European countries, migrant children had a lower risk of overall adverse birth outcome [OR 0.84 (95% CI 0.75–0.94)] and preterm birth [OR 0.81 (95% CI 0.71–0.92)]. All the studies targeting birth outcomes were performed among international migrant children and the effect of domestic migration on birth outcomes cannot be unexplored. Sensitivity analysis of excluding studies with quality score less than five did not alter the above results (Appendix).

**Table 2:** The subgroup analyses by study region.

**Health outcomes**	**European country**		**Non-European country**	
	**Pooled OR (95%CI)**	**Heterogeneity (I2)**	**Pooled OR (95%CI)**	**Heterogeneity (I2)**
**Birth outcome**	0.95(0.90, 1.02)	68.5%	0.84 (0.75, 0.94)	76.7
LBW	0.89 (0.84, 0.94)	73.2%	0.83 (0.65, 1.05)	79.8
HBW	1.11 (0.85,1.45)	69.3%	—	
Preterm birth	0.96 (0.88, 1.04)	75.0%	0.81 (0.71, 0.92)	80.4
**Nutrition**	1.51 (1.29, 1.78)	71.1%	0.98 (0.81, 1.17)	81.7%
Overweight/obesity	1.62 (1.39, 1.90)	68.8%	0.86 (0.68, 1.09)	80.3%
Underweight	0.76 (0.57, 1.01)	0%	—	
Anemia	—		1.37 (1.01, 1.87)	73.6%
**Physical health**	1.48 (1.02, 2.14)	79.1%	1.20 (1.03, 1.41)	74.1%
Oral disease	2.59 (2.10, 3.20)	62.1%	—	
Gastrointestinal disease	—		1.48 (0.94, 2.34)	86.5%
Respiratory disease	0.93 (0.57, 1.54)	78.2%	0.85 (0.57, 1.27)	77.4%
Allergic disease	0.62 (0.51, 0.76)	0%	1.30 (0.77, 2.19)	80.7%
Congenital disease	—		1.32 (0.98, 1.78)	81.1%
**Mental health**	1.17 (0.88, 1.57)	68.4%	1.32 (1.14, 1.52)	49.5%
Depression	—		1.17 (1.09, 1.26)	0%
ADHD	0.73 (0.16, 3.38)	78.8%	—	
ASD	0.93 (0.78, 1.12)	71.8%	—	
Schizophrenia	1.90 (1.75, 2.06)	0%	—	
Suicide attempt	—		—	
**Death**	1.23 (1.13, 1.34)	70.2%	0.97 (0.87, 1.09)	75.9%
Fetal death	1.33 (1.12, 1.34)	67.3%	1.07 (0.96, 1.19)	67.45
Perinatal death	1.25 (1.04, 1.50)	60.0%	—	
Neonatal death	1.20 (1.06, 1.35)	30.7%	1.06 (1.02, 1.10)	0%
Post-neonatal death	1.00 (0.80, 1.25)	0%	0.88 (0.66, 1.16)	66.4%
Infant death	1.08 (0.82, 1.41)	42.3%	0.83 (0.53, 1.29)	74.3%
**Substance use**	0.83 (0.54, 1.27)	78.5%	—	

*LBW, low birth weight; HBW, high birth weight; ADHD, attention deficit hyperactivity disorder; ASD, autistic spectrum disorder. “—” indicate the studies included in the specific outcome no more than one. The pooled OR with 95%CI for the risk of health outcome among subgroup using random-effects model*.

### Nutrition

Migrant children had an increased risk of malnutrition [OR 1.26 (95% CI 1.11–1.44)], including higher risk of overweight/obesity [OR 1.33 (95% CI 1.13–1.57)] and iron-deficiency anemia [OR 1.37 (95% CI 1.01–1.87)]; while no difference was identified regarding underweight [OR 0.90 (95% CI 0.77–1.04)] between migrant and non-migrant children (Figure 2B). Heterogeneity between the estimates was low for underweight and high for overweight/obesity. Subgroup analyses by region (Table 2) revealed that migrant children in European countries had a significantly increased risk of malnutrition [OR 1.51 (95% CI 1.29–1.78)] such as overweight/obesity [OR 1.62 (95% CI 1.39–1.90)], while no significant differences were found between migrant children and native ones in the non-European countries. We also explored the effect of migration way on children's nutrition, which showed that international migrant children had an increased risk of overweight/obesity than non-migrant children [OR 1.47 (95% CI 1.28–1.68)], but the result was opposite for internal migrant children [OR 0.67 (95% CI 0.60–0.74)]. When the studies with quality score less than five were excluded, the risk of malnutrition was not altered (Appendix).

### Physical Health

Migrant children had a significantly increased risk of poor physical health [OR 1.34 (95% CI 1.11–1.61)] compared with non-migrant children, including higher risk of oral disease [OR 2.56 (95% CI 2.11–3.11)] and gastrointestinal disease [OR 1.56 (95% CI 1.18–2.07)] (Figure 2C). Although high statistical heterogeneity was identified across the selected physical health outcomes, a reduction trend was found by using subgroup and sensitivity analysis. Subgroup analyses by region (Table 2) suggested that migrant children had poorer physical health than non-migrant children both in the European countries [OR 1.48 (95% CI 1.02–2.14)] and non-European countries [OR 1.20 (95% CI 1.03–1.41)]. The insufficient number of studies did not allow for analyses of the risk of physical health outcomes among internal migrant children. Sensitivity analyses by excluding studies of quality score less than five did not change the results related to physical health outcomes (Appendix).

### Mental Health

Migrant children had a marginally higher risk of psychological problems [OR 1.24 (95% CI 1.00–1.52)] than the controls, including higher risk of depression [OR 1.29 (95% CI 1.00–1.65)], schizophrenia [OR 1.79 (95% CI 1.50–2.14)], and suicide attempt [OR 1.31 (95% CI 1.10–1.56)] (Figure 2D). Statistical heterogeneity across the mental health outcomes was moderate between estimates. Subgroup analyses by region (Table 2) showed that migrant children in European had an increased risk of schizophrenia; while in non-European countries had higher risk of depression. Given the limited number of studies on internal migrant children, we did not assess the effect of migration way on the risk of mental health outcomes. Sensitivity analyses by excluding studies with quality score less than five did not change the mental health outcomes (Appendix).

### Deaths

All the studies on mortality focused on international migrant children. Migrant children were at a higher risk of death than the controls [OR 1.11 (95% CI 1.01–1.21)], including fetal death [OR 1.24 (95% CI 1.07–1.45)], perinatal death [OR 1.25 (95% CI 1.04–1.50)], and neonatal death [OR 1.10 (95% CI 1.02–1.19)] (Figure 2E). Statistical heterogeneity between estimates varied substantially across death outcomes, with the exception of neonatal death. Subgroup analyses by region (Table 2) on fetal death [OR 1.33 (95% CI 1.12–1.34)], perinatal death [OR 1.25 (95% CI 1.04–1.50)], and neonatal death [OR 1.20 (95% CI 1.06–1.35)] indicated a higher risk for migrant children in European countries than for non-migrant children, but not in the non-European countries, with the exception of neonatal death. The insufficient number of studies did not allow for analyses of the risk of death among internal migrant children. Sensitivity analyses did not change the above results (Appendix).

### Substance Use

No significant differences were found in the risk of substance use [OR 0.83 (95% CI 0.54–1.27)], including alcohol, tobacco, and cannabis use among migrant children compared with non-migrant children (Figure 2F). The above results did not change after sensitivity analyses (Appendix). Given the studies included in substance use were all conducted in the European countries, subgroup analyses by region did not performed. Also, the effect of migration type on substance use did not conducted due to the limited available studies.

The Begg's and Egger's tests indicated no significant publication bias among the included studies in six health outcomes (all *P*_Begg′sTest_ >0.05 and *P*_Egger′sTest_ >0.05).

Meta-regression analyses showed that the sample size, study design, publish year, and study region had effects on physical health outcome (β = 0.557, *SE* = 0.254, *P* = 0.043; β = 0.821, *SE* = 0.281, *P* = 0.010; β = 0.430, *SE* = 0.159, *P* = 0.015; β = 0.498, *SE* = 0.157, *P* = 0.006; respectively), while had no effects on birth outcome and physical health outcome (all *P* > 0.05). Additionally, the effect of study region on nutrition outcome (β = 0.597, *SE* = 0.209, *P* = 0.008) and publish year on mental health outcome (β = −0.557, SE = 0.228, *P* = 0.027) were also observed.

## Discussion

Our findings demonstrated that migrant children tend to have overall worse health outcomes than non-migrant children. Compared with the controls, migrant children had an increased risk of malnutrition (e.g., overweight/obesity and anemia), poor physical health (oral diseases and gastrointestinal diseases), mental disorder (e.g., depression, schizophrenia, and suicide attempt), and death (fetal death, perinatal death, and neonatal death). The beneficial health effects were observed in birth outcomes such as lower risk of LBW and preterm birth.

### The Healthy Migration Effect Does Not Necessarily Exist in Migrant Children Although Superior Birth Outcome Was Observed

“The immigrant paradox” has been reported in studies targeting the adult migration population. Despite the average lower socio-economic status of migrants and their inferior access to healthcare, adult migrants in advanced societies are generally healthier than the natives in the host country (17). The healthy immigrant effect was also reported in some health outcomes in children upon their birth or arrival. A review on international migrants in Spain suggested that children with migrant mothers have superior birth outcomes, such as a lower incidence of LBW and preterm birth than the natives (108), which is consistent with the finding of our meta-analysis. Specific factors such as mother's healthier migrant lifestyles and the cultural heritages of the migrant countries (e.g., lower rates of smoking and alcohol consumption) may partially explain the phenomenon (109). Another explanation is the selective migration hypothesis that healthier and/or wealthier women may choose to migrate to richer countries where they can have better birth outcomes (31). However, the notion that the health effect does not apply to all migrants is a subject of debate. Due to the limited generalizability, the immigrant paradox may be better conceptualized as outcome-specific with consideration of such relevant factors as immigrants' ethnicity, length of residence (10), nativity, and age at arrival (110). This meta-analysis suggests that the immigrant paradox does not necessarily exist among children in multiple outcomes. Migrant children have an overall poorer health status, especially in overweight/obesity, mental disorder, poorly physical health, and mortality.

### Migrant Children Have Higher Risk of Developing Malnutrition, Especially Being Overweight/Obesity

As reported, migrant children adhered poorly to health diet recommendations for vegetable consumption and more likely to consume sweet and soft drinks than did the native residents, which is a driver factor for obesity (111). Our meta-analysis indicated an increased risk of overweight/obesity in migrant children, especially in those who migrated to European countries with high incomes, which were consistent with the concept that migration to developed countries may develop to be overweight and obesity (112). The increased risk of obesity among migrant children can be caused by alterations in dietary intake and adopting “unhealthier” practices of the host nations (113), including increased saturated fat and carbohydrate consumption. Eating disorder among migrants may be associated with stress during acculturation compounded by pressure to adapt to new cultural body shape norms (114). Additionally, children within lower income migrant families may easily exposed to more processed and energy-dense foods because they are cheaper and quicker to prepare (111). Moreover, alterations in physical activity, a more sedentary way of life, and lower sleep duration among migrant children (115), are also important drivers for overweight and obesity (116). Our study also suggested that international migrant children had a higher risk of overweight/obesity, but the opposite result was observed among children migrating within the country. As we known, international migrants from low-middle income countries to high income countries were more likely to adopt the above-mentioned westernized lifestyle and unhealthy dietary habits (e.g., high energy, sugar, and fat intake) which were the key risk of overweight/obesity. While the rural-to-urban migrant children in India and China usually live in lower socioeconomic families and may less likely to access to more other foods compared to urban children. Yet, the prevalence of overweight/obesity of rural-to-urban migrant children is increasing gradually in recent year, which need to be of concern.

### Psychological Wellbeing Is Also One of Concerns in the Broader Population of Migrant Children

Our study found that migrant children have poorer mental health than their indigenous peers, including higher risk of depression, suicide attempt, and schizophrenia. In general, stress, anxiety and depression in migrant children are strongly influenced by psychological adaption within the host country (117). Acculturation stress which refers to the potential challenges migrants face when they negotiate differences between their home and host cultures (118) increases the risks of various mental health problems among immigrant adolescents, including withdrawn, somatic, and anxious/depressed symptoms (119). Such stress arises from multiple aspects of the acculturation process, including learning new and sometimes confusing cultural rules and expectations, dealing with prejudice and discrimination, and managing the overarching conflict between maintaining elements of the old culture while incorporating those of the new (120). By the way, the publication year of the included studies had effect on migrant children's mental health in our meta-regression analysis, this may be connected with the phenomenon that increasing number of researches focused on mental health were appeared in a decade year with the progress of globalization.

### Poorly Experience of Health Is Not Uncommon Among Migrant Children

As reported that migrant children have high levels of ill health and unmet healthcare needs (121). In our study, migrant children have increased risk of mortality such as fetal death, perinatal death, and neonatal death, as well as worse physical health such as oral diseases and gastrointestinal diseases including diarrhea. The limited access to health service and insurance are the most challenging barriers for this situations (122). Experiences of health services are often unsatisfactory for migrant children, such as difficulties and delay in registering with the General medical Practitioners, difficulties securing medical appointments and missed follow-up appointments (121). Studies suggests that migrant children are four times as likely to be uninsured as native children (7). Moreover, access to health care may also be limited by their parents' knowledge and healthcare awareness, and language and cultural barriers (123–125). Additionally, the effects of poverty on access to health insurance and healthcare appear to be the strongest (7). Children from a migrant household are more likely to live in poverty than children from a non-migrant household. For US migrant families, children in poorer families were nearly twice as likely to have not visited a dentist and to lack a usual source of sick care, and 50% were more likely not to have visited a doctor in the previous year (7).

### Actions Are Required to Address the Health Inequity Among These Populations

To date, monitoring migrant health is among the key priorities of the International Organization for Migration, and a set of actions have been taken to monitor migrants' health-seeking behaviors, access to and utilization of health services, and to increase the collection of data related to health status and outcomes of migrants (1). However, strategies specially designed to improve the birth and health status of migrant children remain insufficient. Through the United Nations 2030 Agenda for Sustainable Development, countries worldwide have pledged to take actions to achieve the Sustainable Development Goals, including Goal 3 of good health and wellbeing and Goal 10 of reduced inequalities. Yet, the health inequalities are still prevalent. Poor health outcomes are secondary to system (e.g., long wait times between making appointments and seeing health professionals, and the long wait times at health facilities), financial, and language and cultural barriers (126). Addressing those barriers should be prioritized if the health status of migrants is to be improved. First, developing migrant-sensitive health systems and ensuring that health services are delivered to migrant children in a culturally and linguistically appropriate way, and enforce laws and regulations that prohibit discrimination. Second, adopting measures to improve the ability of health systems to deliver migrant inclusive services and programmes in a comprehensive, coordinated and financially sustainable way. Third, identifying good practices in monitoring migrant children's health and mapping policy models that facilitate equitable access to health care (1).

### Strength and Limitations

The comprehensive scope of this meta-analysis is a strength since evidence across multiple health outcomes and with low publication bias. However, our study has several limitations. First, our original systematic search included literature published up to January 30, 2021, and thus newer studies may draw different conclusions. Second, statistical heterogeneity was moderate high in this meta-analysis, which did not significantly decrease after subgroup-analyses. Yet, meta-regression indicated that the sample size, study design, publish year, or study region had effects on multiple health outcomes, which may partly explain the source of high heterogeneity. Similarly, high heterogeneity was identified in a systematic review and meta-analyses of the health impacts of parental migration on left-behind children (127). Third, most of the included studies in our meta-analysis were from European countries, focused on international migration and were cross-sectional, which means temporal causal inference is limited and might not generalized. Fourth, the studies with forced migrant and unaccompanied children were excluded, which might have underestimated the health status of the migrant children. Fifth, we only included studies published in English language, the non-English studies with internal migrant children especially in Chinese publications might have been excluded. Last but not the least, we were unable to explore the effect of socioeconomic status, origin country, migrant generation (e.g., the first-generation and second-generation migration) and length of residence in the host country on the health outcomes of migrant children due to the unavailability of this information, which might contribute to the migration paradox.

## Conclusion

Children migrating with parents have higher risk of poor health outcomes such as malnutrition, physical diseases, mental disorder, and death than the host populations. The healthy migrant paradox does not necessary exist among children in multiple outcomes. Interventions that support migrants are urgently needed to prevent long-term negative effects on their health and development.

## Data Availability Statement

The original contributions presented in the study are included in the article/Supplementary Material, further inquiries can be directed to the corresponding author.

## Author Contributions

RC developed the study and oversaw its implementation, analyzed the data, and wrote the manuscript. RC, CL, HQ, and YZ did review activities, consisting of searches, study selection, data extraction, and quality assessment. JZ conceptualized and designed the study, coordinated, supervised data collection, and critically reviewed the manuscript for important intellectual content. All authors reviewed the study findings, read, and approved the final version before submission.

## Funding

This study was supported by the Fundamental Research Funds for the Central Universities (HUST: 2020WKZDJC012) and by the Research and Publicity Department of China Association for Science and Technology (20200608CG111312).

## Conflict of Interest

The authors declare that the research was conducted in the absence of any commercial or financial relationships that could be construed as a potential conflict of interest.

## Publisher's Note

All claims expressed in this article are solely those of the authors and do not necessarily represent those of their affiliated organizations, or those of the publisher, the editors and the reviewers. Any product that may be evaluated in this article, or claim that may be made by its manufacturer, is not guaranteed or endorsed by the publisher.
