# Supplementary material for: Birth and Health Outcomes of Children Migrating With Parents: A Systematic Review and Meta-Analysis
Source: Front Pediatr. 2022 Jul 13;10:810150. doi: 10.3389/fped.2022.810150 (PMC9326113; doi:10.3389/fped.2022.810150)
Supplement: Supplementary file 1 [file Table_1.DOC]

**Supplementary materials**

**Birth and health outcomes of children migrating with parents:**

**a systematic review and meta-analysis**

Ruixia Chang, Chunan Li, Ya Zhang, Haiqin Qi, Jianduan Zhang

Department of Maternal and Child Health, School of Public Health, Tongji Medical College, Huazhong University of Science and Technology, 13 Hangkong Road, Wuhan, 430030, Hubei, China.

**^*^**corresponding author. Department of Maternal and Child Health, School of Public Health, Tongji Medical College, Huazhong University of Science and Technology, 13 Hangkong Road, Wuhan, China. Email: [1999020628@hust.edu.cn](mailto:1999020628@hust.edu.cn).

**Contents**

Preferred reporting items for systematic reviews and meta-analysis (PRISMA) guidelines------------------------------2

Inclusion and exclusion criteria----------------------------------------------------------------------------------------------------5

Full search strategy------------------------------------------------------------------------------------------------------------------7

Quality assessment of studies included-------------------------------------------------------------------------------------------8

Sensitivity analysis-----------------------------------------------------------------------------------------------------------------13

| **Supplement table 1 Preferred reporting items for systematic reviews and meta-analysis (PRISMA) guidelines** | | | |
| --- | --- | --- | --- |
| **Section/topic** | **#** | **Checklist item** | **Reported on page #** |
| **TITLE** | | |  |
| Title | 1 | Identify the report as a systematic review, meta-analysis, or both. | 1 |
| **ABSTRACT** | | |  |
| Structured summary | 2 | Provide a structured summary including, as applicable: background; objectives; data sources; study eligibility criteria, participants, and interventions; study appraisal and synthesis methods; results; limitations; conclusions and implications of key findings; systematic review registration number. | 1,2 |
| **INTRODUCTION** | | |  |
| Rationale | 3 | Describe the rationale for the review in the context of what is already known. | 2,3 |
| Objectives | 4 | Provide an explicit statement of questions being addressed with reference to participants, interventions, comparisons, outcomes, and study design (PICOS). | 2,3 |
| **METHODS** | | |  |
| Protocol and registration | 5 | Indicate if a review protocol exists, if and where it can be accessed (e.g., Web address), and, if available, provide registration information including registration number. | 6 |
| Eligibility criteria | 6 | Specify study characteristics (e.g., PICOS, length of follow-up) and report characteristics (e.g., years considered, language, publication status) used as criteria for eligibility, giving rationale. | 4, Appendix |
| Information sources | 7 | Describe all information sources (e.g., databases with dates of coverage, contact with study authors to identify additional studies) in the search and date last searched. | 4 |
| Search | 8 | Present full electronic search strategy for at least one database, including any limits used, such that it could be repeated. | 4, Appendix |
| Study selection | 9 | State the process for selecting studies (i.e., screening, eligibility, included in systematic review, and, if applicable, included in the meta-analysis). | 4, Figure 1 |
| Data collection process | 10 | Describe method of data extraction from reports (e.g., piloted forms, independently, in duplicate) and any processes for obtaining and confirming data from investigators. | 5 |
| Data items | 11 | List and define all variables for which data were sought (e.g., PICOS, funding sources) and any assumptions and simplifications made. | 5, Appendix |
| Risk of bias in individual studies | 12 | Describe methods used for assessing risk of bias of individual studies (including specification of whether this was done at the study or outcome level), and how this information is to be used in any data synthesis. | 5,6 |
| Summary measures | 13 | State the principal summary measures (e.g., risk ratio, difference in means). | 6 |
| Synthesis of results | 14 | Describe the methods of handling data and combining results of studies, if done, including measures of consistency (e.g., I^2^) for each meta-analysis. | 6 |

| Risk of bias across studies | 15 | Specify any assessment of risk of bias that may affect the cumulative evidence (e.g., publication bias, selective reporting within studies). | 6 |
| --- | --- | --- | --- |
| Additional analyses | 16 | Describe methods of additional analyses (e.g., sensitivity or subgroup analyses, meta-regression), if done, indicating which were pre-specified. | 6 |
| **RESULTS** | | |  |
| Study selection | 17 | Give numbers of studies screened, assessed for eligibility, and included in the review, with reasons for exclusions at each stage, ideally with a flow diagram. | 6,7, Figure1, appendix |
| Study characteristics | 18 | For each study, present characteristics for which data were extracted (e.g., study size, PICOS, follow-up period) and provide the citations. | 7, Appendix |
| Risk of bias within studies | 19 | Present data on risk of bias of each study and, if available, any outcome level assessment (see item 12). | 8, Appendix |
| Results of individual studies | 20 | For all outcomes considered (benefits or harms), present, for each study: (a) simple summary data for each intervention group (b) effect estimates and confidence intervals, ideally with a forest plot. | 7-12, Figure 2A-F |
| Synthesis of results | 21 | Present results of each meta-analysis done, including confidence intervals and measures of consistency. | 7-12, Figure 2A-F |
| Risk of bias across studies | 22 | Present results of any assessment of risk of bias across studies (see Item 15). | 8, Appendix |
| Additional analysis | 23 | Give results of additional analyses, if done (e.g., sensitivity or subgroup analyses, meta-regression [see Item 16]). | 7-12, Appendix |
| **DISCUSSION** | | |  |
| Summary of evidence | 24 | Summarize the main findings including the strength of evidence for each main outcome; consider their relevance to key groups (e.g., healthcare providers, users, and policy makers). | 11 |
| Limitations | 25 | Discuss limitations at study and outcome level (e.g., risk of bias), and at review-level (e.g., incomplete retrieval of identified research, reporting bias). | 15 |
| Conclusions | 26 | Provide a general interpretation of the results in the context of other evidence, and implications for future research. | 16 |
| **FUNDING** | | |  |
| Funding | 27 | Describe sources of funding for the systematic review and other support (e.g., supply of data); role of funders for the systematic review. | 16 |

*From:*  Moher D, Liberati A, Tetzlaff J, Altman DG, The PRISMA Group (2009). Preferred Reporting Items for Systematic Reviews and Meta-Analyses: The PRISMA Statement. PLoS Med 6(7): e1000097. doi:10.1371/journal.pmed1000097

For more information, visit: **www.prisma-statement.org**.

**Inclusion and exclusion criteria**

| **Supplement table 2 Health outcomes included in the systematic review** | | | |
| --- | --- | --- | --- |
| Area of health | Outcomes | Study n | Outcome measures |
| Birth outcome | Low birth weight (LBW) | 24 | - Clinical outcomes: anthropometric measurements, records |
|  | High birth weight (HBW) | 12 |  |
|  | Preterm birth | 19 |  |
| Nutrition | Overweight/obesity | 23 | - Clinical outcomes: anthropometric measurements, serum sample |
|  | Underweight | 3 |  |
|  | Anemia | 3 |  |
| Physical health | Oral disease | 9 | - Clinical outcomes: clinical assessment, self-reports of physician/clinician diagnosis - Self-reported symptoms e.g. of diarrhea |
|  | Gastrointestinal disease (gastroenteritis, diarrhea) | 3 |  |
|  | Respiratory disease (pneumonia, asthma) | 12 |  |
|  | Allergic disease (eczema, food allergy) | 6 |  |
|  | Congenital disease (heart defect, neural tube defect) | 4 |  |
| Mental health | Depression | 4 | - Clinical outcomes: clinical assessment (ICD9/ICD10) - Self-reported symptoms: validated mental health screening tools |
|  | Attention deficit hyperactivity disorder (ADHD) | 3 |  |
|  | Autistic spectrum disorder (ASD) | 4 |  |
|  | Schizophrenia | 3 |  |
|  | Suicide attempt | 2 |  |
| Death | Fetal death (more than 500 g at 22 or more weeks of gestation) | 7 | - clinical outcomes: clinical assessment, birth and death certificates |
|  | Perinatal death (from 22 weeks of gestation until 7 days after birth) | 5 |  |
|  | Neonatal death (0-28 days) | 7 |  |
|  | Post-neonatal (28-364 days) | 5 |  |
|  | Infant death (0-11months) | 4 |  |
| Substance use | Tobacco, Alcohol, Cannabis | 8 | - Self-reported symptoms: screening tools, reported substance use |

**Inclusion codes**

1.Study is a cohort, case-control, or cross-sectional

2.Participates children aged < 19 years

3.Migrant children were those migrating with parents internationally (across the country border, e.g.first-generation migrations and second-generation migrations) and internally (within the country, e.g. rural to urban migration)

4.Control of children were native ones

5.Assess any of the following outcomes: birth outcome, nutrition, mental health, physical health, death, substance use.

**Exclusion codes**

1. Study on refugee children due to armed conflict, disasters, political, religious or ethnic persecution
2. Study language was not in English
3. Simple size less than 500
4. Reviews
5. Conference proceedings
6. Protocol for a study not meeting the inclusion criteria
7. Cannot locate full text
8. Ongoing studies

**Full search strategy**

**Supplement table 3 The full search strategy of the meta-analysis**

| Concept 1: children |
| --- |
| 1. (((child*[Title/Abstract] OR infant[Title/Abstract] OR adolescen*[Title/Abstract] OR "young adult*"[Title/Abstract] OR teen*[Title/Abstract] OR "young person*"[Title/Abstract] OR juvenile[Title/Abstract] OR boy[Title/Abstract] OR girl[Title/Abstract] OR youth[Title/Abstract] OR pupil*[Title/Abstract] OR student*[Title/Abstract] OR newborn[Title/Abstract] OR baby[Title/Abstract] OR child[Title/Abstract] OR infant[Title/Abstract] OR infant[Title/Abstract] OR newborn[Title/Abstract] OR child[Title/Abstract] OR preschool[Title/Abstract] OR adolescent[Title/Abstract])) |
| Concept 2: parent |
| 1. (parent*[Title/Abstract] OR mother[Title/Abstract] OR father[Title/Abstract] OR guardian*[Title/Abstract] OR caregiver[Title/Abstract]) |
| Concept 3: migration |
| 1. (Emigration[Title/Abstract] OR immigration[Title/Abstract] OR migration[Title/Abstract] OR migrants[Title/Abstract] OR emigrants[Title/Abstract] OR migrate[Title/Abstract] OR immigrate[Title/Abstract] OR migrant[Title/Abstract] OR emigrant[Title/Abstract])) |
| Combining all concepts |
| 1. 1 and 2 and 3 |

Quality assessment

| **Supplement table 4 Template data extraction sheet for quality assessment (Newcastle Ottawa Scale)** | |
| --- | --- |
| Item number | Risk of Bias Assessment |
|  | 1. POPULATION |
| 1 | Were migrant children well defined? |
| 2 | Were children in the control group well described/defined? |
|  | 1. SELECTION |
| 3 | Was selection bias minimized? |
|  | 1. OUTCOME |
| 4 | Were the outcome measures and procedures reliable? |
| 5 | Were all outcome measures reported? |
|  | D. ANALYSIS |
| 6 | Was the study sufficiently powered to detect a difference, if one exists? |
| 7 | Were the statistical analyses clear and presented with a measure of precision? |
| 8 | Was incomplete outcome data adequately addressed? |
| 9 | Were potential confounders adequately addressed? |

| **Supplement table 5 Quality assessment of studies included in the systematic review (N=98)** | | | | | | | | | | | |
| --- | --- | --- | --- | --- | --- | --- | --- | --- | --- | --- | --- |
| First author | Year | Definition of cases | Definition of controls | Selection bias | Reliability of outcome measures | Outcome measure reporting | Power | Statistical analyses | Incomplete outcome data | Potential confounders | Overall assessment |
| Kana [5] | 2019 | 0 | 1 | 0 | 0 | 1 | 1 | 0 | 0 | 1 | 4 |
| Gillet [9] | 2014 | 0 | 0 | 0 | 0 | 1 | 1 | 1 | 0 | 1 | 4 |
| Cebolla-Boado [17] | 2016 | 1 | 1 | 0 | 1 | 1 | 1 | 1 | 0 | 1 | 7 |
| Forna [18] | 2003 | 0 | 0 | 1 | 1 | 1 | 1 | 1 | 1 | 1 | 7 |
| Sandra [19] | 2015 | 0 | 0 | 1 | 1 | 1 | 1 | 1 | 0 | 1 | 6 |
| Besharat [20] | 2014 | 1 | 0 | 0 | 0 | 1 | 0 | 1 | 0 | 1 | 4 |
| Racape [21] | 2016 | 0 | 0 | 0 | 1 | 1 | 1 | 1 | 0 | 0 | 4 |
| Lehti [22] | 2013 | 0 | 0 | 0 | 1 | 1 | 1 | 1 | 0 | 0 | 4 |
| Milewski [23] | 2014 | 1 | 1 | 1 | 1 | 1 | 0 | 1 | 0 | 0 | 6 |
| Ratnasiri [24] | 2020 | 1 | 1 | 1 | 1 | 0 | 1 | 1 | 0 | 1 | 7 |
| Nancy [25] | 2014 | 0 | 0 | 0 | 1 | 1 | 0 | 1 | 0 | 0 | 3 |
| Castello [26] | 2012 | 0 | 0 | 1 | 1 | 1 | 1 | 1 | 0 | 1 | 6 |
| Juarez [27] | 2014 | 1 | 0 | 1 | 1 | 1 | 1 | 1 | 1 | 1 | 8 |
| Fuster [28] | 2014 | 0 | 0 | 0 | 0 | 1 | 1 | 1 | 0 | 0 | 3 |
| Racape [29] | 2010 | 0 | 0 | 0 | 1 | 1 | 0 | 1 | 0 | 0 | 3 |
| Glick [30] | 2009 | 1 | 1 | 1 | 1 | 1 | 1 | 1 | 1 | 0 | 8 |
| Farre [31] | 2013 | 1 | 0 | 1 | 1 | 1 | 1 | 1 | 0 | 1 | 7 |
| Nancy S [32] | 2015 | 1 | 1 | 1 | 1 | 0 | 1 | 1 | 0 | 1 | 7 |
| Lehti [33] | 2016 | 1 | 1 | 0 | 1 | 1 | 1 | 1 | 0 | 1 | 7 |
| Mika [34] | 2003 | 1 | 1 | 1 | 1 | 1 | 1 | 1 | 0 | 1 | 8 |
| Madan [35] | 2006 | 0 | 0 | 0 | 0 | 1 | 1 | 1 | 0 | 0 | 3 |
| Auger [36] | 2008 | 1 | 0 | 1 | 1 | 1 | 1 | 1 | 0 | 1 | 7 |
| Bastola [37] | 2020 | 1 | 1 | 0 | 1 | 1 | 0 | 1 | 1 | 1 | 7 |
| Marcon [38] | 2011 | 1 | 1 | 0 | 0 | 1 | 0 | 1 | 0 | 0 | 4 |
| Besharat [39] | 2017 | 1 | 1 | 0 | 1 | 1 | 0 | 1 | 1 | 1 | 7 |
| Reeske [40] | 2013 | 1 | 1 | 1 | 0 | 1 | 1 | 1 | 0 | 0 | 6 |
| Choi [41] | 2019 | 1 | 1 | 0 | 1 | 1 | 1 | 1 | 1 | 1 | 8 |
| Essen [42] | 2000 | 1 | 1 | 0 | 1 | 1 | 1 | 1 | 1 | 0 | 7 |
| Vik [43] | 2019 | 1 | 1 | 0 | 1 | 1 | 1 | 1 | 1 | 1 | 8 |
| Kumar [44] | 2016 | 1 | 1 | 0 | 0 | 0 | 0 | 1 | 0 | 0 | 3 |
| Liu [45] | 2016 | 0 | 0 | 1 | 1 | 1 | 1 | 1 | 1 | 1 | 7 |
| Ji [14] | 2016 | 1 | 1 | 1 | 0 | 1 | 1 | 1 | 0 | 1 | 7 |
| Lin [46] | 2011 | 1 | 1 | 0 | 1 | 1 | 1 | 1 | 0 | 1 | 7 |
| De [47] | 2018 | 0 | 0 | 0 | 1 | 1 | 0 | 1 | 0 | 0 | 3 |
| Zulfiqar [48] | 2018 | 1 | 1 | 0 | 1 | 1 | 1 | 1 | 0 | 1 | 7 |
| Maximova [13] | 2011 | 1 | 1 | 1 | 1 | 1 | 1 | 1 | 0 | 1 | 8 |
| Lindstrom [49] | 2014 | 1 | 1 | 0 | 1 | 1 | 1 | 1 | 0 | 1 | 7 |
| Esteban 50] | 2014 | 1 | 1 | 1 | 1 | 1 | 1 | 1 | 0 | 1 | 8 |
| Besharat [51] | 2014 | 0 | 0 | 1 | 1 | 1 | 1 | 1 | 1 | 1 | 7 |
| Furthner [52] | 2017 | 0 | 0 | 1 | 1 | 1 | 1 | 1 | 1 | 1 | 7 |
| Burgi [53] | 2010 | 1 | 1 | 0 | 0 | 1 | 0 | 1 | 0 | 0 | 4 |
| Iguacel [54] | 2018 | 1 | 1 | 1 | 0 | 1 | 1 | 0 | 0 | 1 | 6 |
| Khanolkar [55] | 2013 | 1 | 1 | 0 | 0 | 1 | 1 | 1 | 0 | 1 | 6 |
| Thi [56] | 2019 | 1 | 1 | 1 | 1 | 0 | 1 | 1 | 0 | 1 | 7 |
| Will [57] | 2005 | 1 | 0 | 1 | 1 | 1 | 1 | 1 | 1 | 1 | 8 |
| Zhou [58] | 2018 | 1 | 1 | 0 | 1 | 1 | 1 | 1 | 1 | 0 | 7 |
| Meroc [15] | 2019 | 1 | 1 | 1 | 0 | 1 | 1 | 1 | 0 | 1 | 7 |
| Labree [59] | 2015 | 1 | 0 | 1 | 1 | 1 | 1 | 1 | 1 | 0 | 7 |
| Brettschneider [60] | 2011 | 0 | 0 | 1 | 1 | 1 | 1 | 1 | 0 | 1 | 6 |
| Vorwieger [61] | 2018 | 1 | 1 | 0 | 1 | 1 | 1 | 1 | 0 | 0 | 6 |
| Nagel [62] | 2009 | 1 | 1 | 1 | 1 | 1 | 1 | 1 | 0 | 0 | 7 |
| Beyerlein [63] | 2014 | 0 | 1 | 1 | 1 | 1 | 1 | 1 | 1 | 1 | 8 |
| Prusty [64] | 2015 | 1 | 1 | 1 | 1 | 1 | 1 | 1 | 0 | 0 | 7 |
| Saunders [65] | 2016 | 1 | 1 | 0 | 0 | 1 | 1 | 1 | 1 | 1 | 7 |
| Hu [66] | 2014 | 1 | 1 | 1 | 0 | 1 | 1 | 1 | 0 | 1 | 7 |
| Julihn [67] | 2010 | 1 | 1 | 1 | 1 | 1 | 1 | 1 | 0 | 1 | 8 |
| Christensen [68] | 2010 | 0 | 0 | 0 | 0 | 1 | 1 | 1 | 0 | 0 | 3 |
| Van Meijeren [69] | 2019 | 1 | 1 | 0 | 1 | 1 | 1 | 1 | 1 | 1 | 8 |
| Ferrazzano [70] | 2019 | 1 | 1 | 1 | 1 | 1 | 1 | 1 | 0 | 0 | 7 |
| Van der [71] | 2016 | 1 | 1 | 0 | 0 | 1 | 1 | 1 | 1 | 1 | 7 |
| Almerich [72] | 2007 | 0 | 0 | 0 | 0 | 1 | 1 | 0 | 0 | 1 | 3 |
| Bissar [73] | 2014 | 1 | 1 | 1 | 1 | 1 | 1 | 1 | 0 | 1 | 8 |
| Baggio [74] | 2015 | 0 | 0 | 1 | 1 | 1 | 1 | 1 | 1 | 1 | 7 |
| Bardin [75] | 2019 | 1 | 0 | 1 | 1 | 1 | 1 | 1 | 0 | 1 | 7 |
| Charania [76] | 2020 | 1 | 1 | 1 | 1 | 0 | 1 | 1 | 1 | 1 | 8 |
| Li [77] | 2019 | 0 | 0 | 1 | 0 | 1 | 1 | 0 | 0 | 0 | 3 |
| Migliore [78] | 2007 | 0 | 0 | 1 | 0 | 1 | 0 | 1 | 0 | 1 | 4 |
| Keet [79] | 2012 | 0 | 0 | 0 | 1 | 1 | 1 | 1 | 0 | 0 | 4 |
| Svendsen [80] | 2009 | 0 | 0 | 0 | 0 | 1 | 1 | 0 | 0 | 1 | 3 |
| Radhakrishnan [81] | 2019 | 1 | 1 | 1 | 1 | 1 | 1 | 1 | 0 | 1 | 8 |
| Apfelbacher [82] | 2011 | 1 | 1 | 1 | 1 | 1 | 1 | 1 | 0 | 1 | 8 |
| Koplin [83] | 2014 | 0 | 1 | 1 | 1 | 1 | 1 | 1 | 0 | 1 | 7 |
| Ramadhani [804] | 2009 | 1 | 1 | 1 | 1 | 1 | 1 | 1 | 0 | 1 | 8 |
| Kang [85] | 2016 | 0 | 0 | 1 | 1 | 1 | 1 | 1 | 1 | 0 | 6 |
| Velie [86] | 2006 | 1 | 1 | 0 | 0 | 1 | 1 | 1 | 1 | 1 | 7 |
| Kim [87] | 2018 | 1 | 1 | 0 | 0 | 1 | 1 | 1 | 1 | 1 | 7 |
| Fuhrmann [88] | 2014 | 1 | 1 | 1 | 1 | 0 | 1 | 1 | 0 | 0 | 6 |
| Adriaanse [89] | 2014 | 1 | 1 | 1 | 0 | 1 | 1 | 0 | 1 | 1 | 7 |
| Wang [90] | 2017 | 1 | 1 | 1 | 1 | 1 | 1 | 1 | 0 | 1 | 8 |
| Van der [91] | 2013 | 1 | 1 | 1 | 1 | 1 | 1 | 1 | 0 | 1 | 8 |
| Wandell [92] | 2020 | 1 | 0 | 1 | 1 | 1 | 1 | 1 | 0 | 1 | 7 |
| Magnusson [93] | 2012 | 0 | 0 | 0 | 0 | 1 | 1 | 1 | 0 | 0 | 3 |
| Weiser [94] | 2008 | 0 | 0 | 1 | 1 | 1 | 1 | 1 | 1 | 1 | 7 |
| Hjern [95] | 2004 | 1 | 1 | 1 | 1 | 1 | 1 | 1 | 0 | 1 | 8 |
| Pedersen [96] | 2012 | 0 | 0 | 0 | 1 | 1 | 1 | 0 | 0 | 0 | 3 |
| Lu [97] | 2020 | 0 | 0 | 1 | 1 | 1 | 1 | 1 | 1 | 1 | 7 |
| Vazsonyi [98] | 2017 | 1 | 1 | 0 | 0 | 1 | 1 | 1 | 0 | 1 | 6 |
| Villadsen [99] | 2010 | 1 | 1 | 1 | 1 | 0 | 1 | 1 | 0 | 1 | 7 |
| Barona-Vilar [100] | 2014 | 1 | 1 | 1 | 1 | 1 | 1 | 1 | 0 | 0 | 7 |
| Vang [101] | 2016 | 0 | 0 | 1 | 1 | 1 | 1 | 1 | 1 | 1 | 7 |
| Rosenberg [102] | 2002 | 1 | 1 | 1 | 1 | 1 | 1 | 1 | 0 | 0 | 7 |
| Landale [103] | 2006 | 0 | 0 | 0 | 0 | 1 | 1 | 0 | 0 | 1 | 3 |
| Troe [104] | 2007 | 1 | 1 | 1 | 0 | 1 | 1 | 1 | 0 | 1 | 7 |
| Abebe [105] | 2015 | 0 | 0 | 1 | 1 | 0 | 0 | 1 | 0 | 1 | 4 |
| Slonim-Nevo [106] | 2006 | 1 | 1 | 0 | 0 | 0 | 1 | 1 | 0 | 0 | 4 |
| Donath [107] | 2016 | 0 | 0 | 1 | 1 | 1 | 1 | 1 | 1 | 0 | 6 |

Notes: 0 represents high or unclear risk of bias, 1 represents low risk of bias. The quality assessment was based on an adapted version of the Newcastle Ottawa Scale incorporating items from the national institute for clinical excellence quality appraisal. Studies with high or unclear risk of bias across five or more domains were defined as being at high risk of bias overall.

**Sensitivity analyses (excluding studies with quality score＜5)**


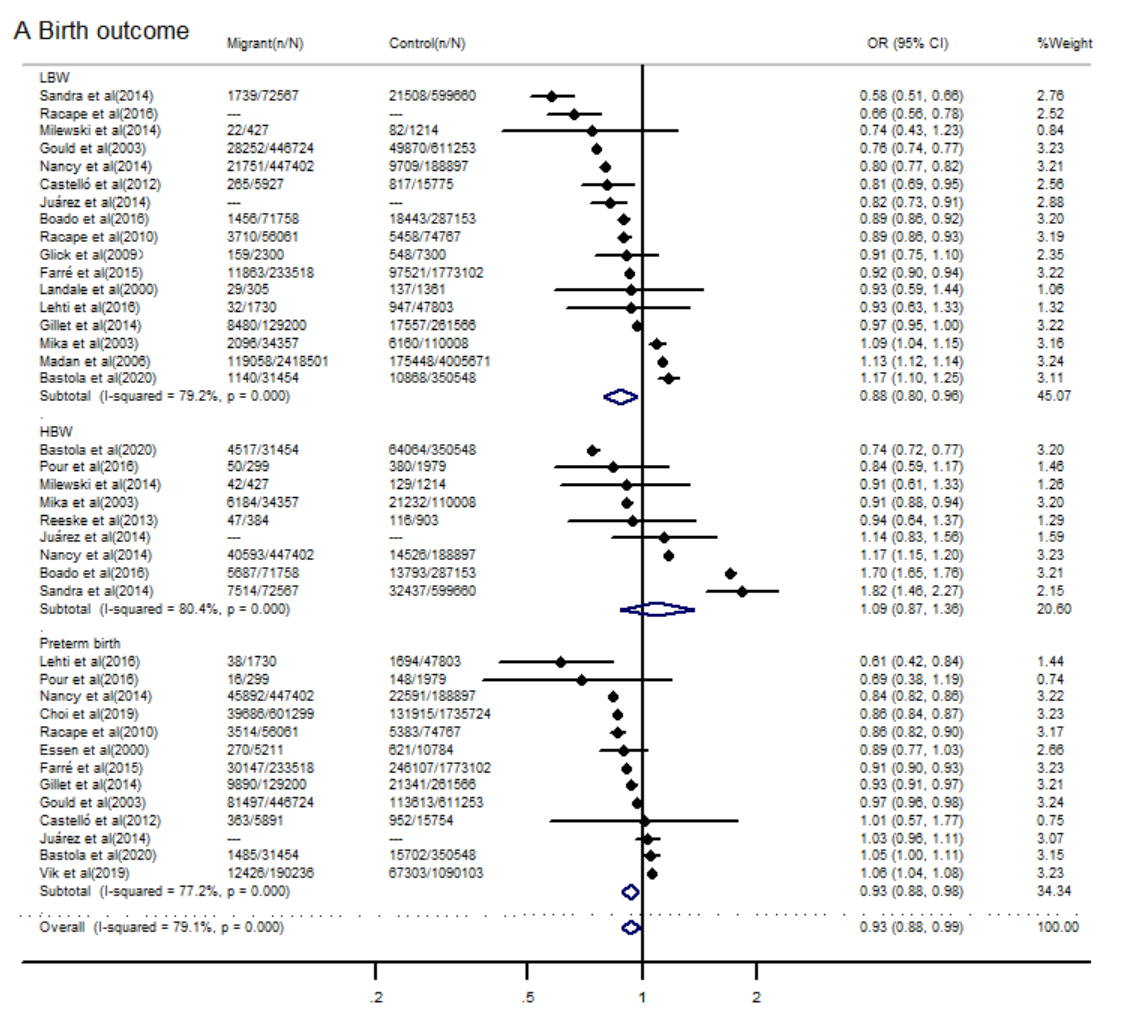


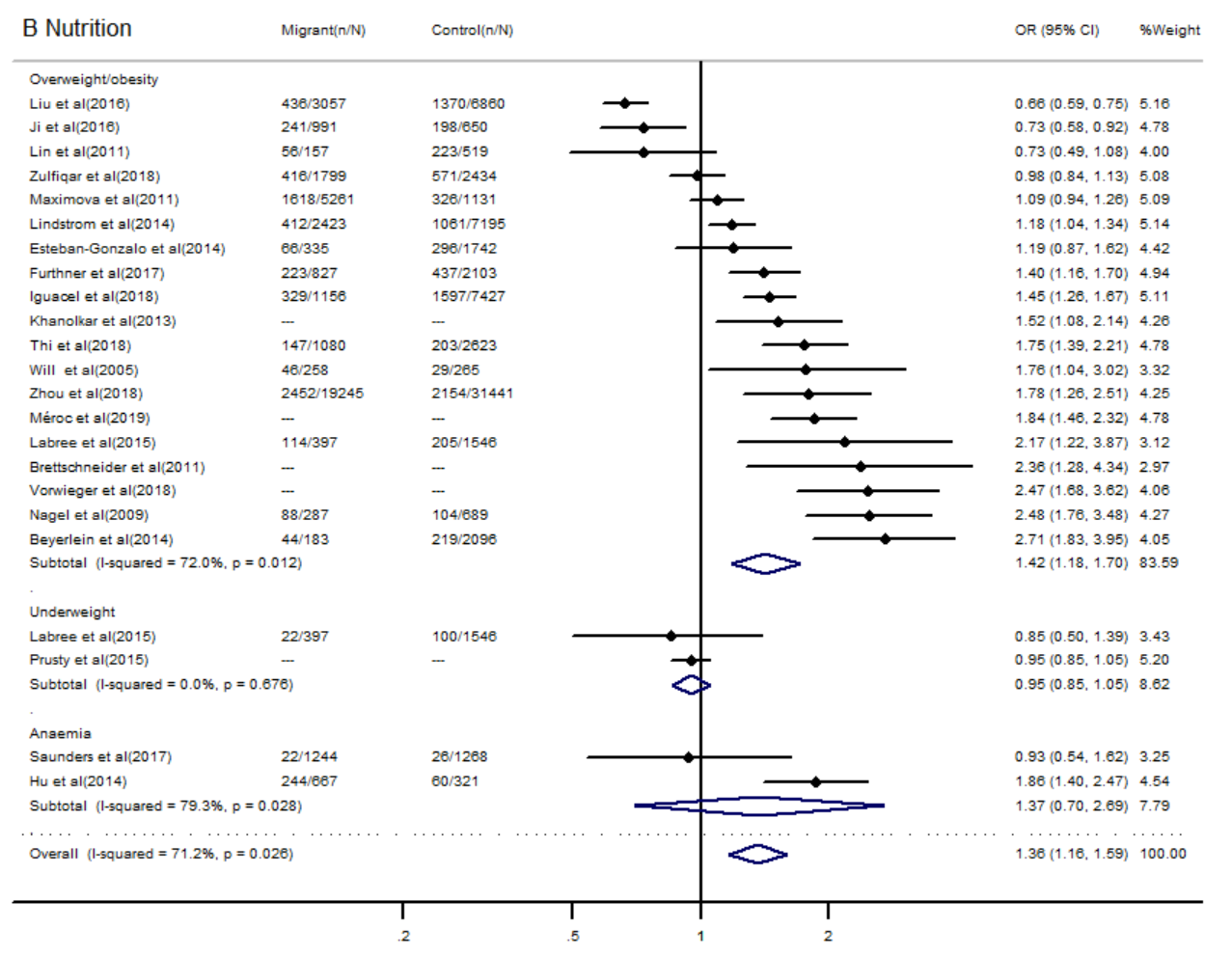


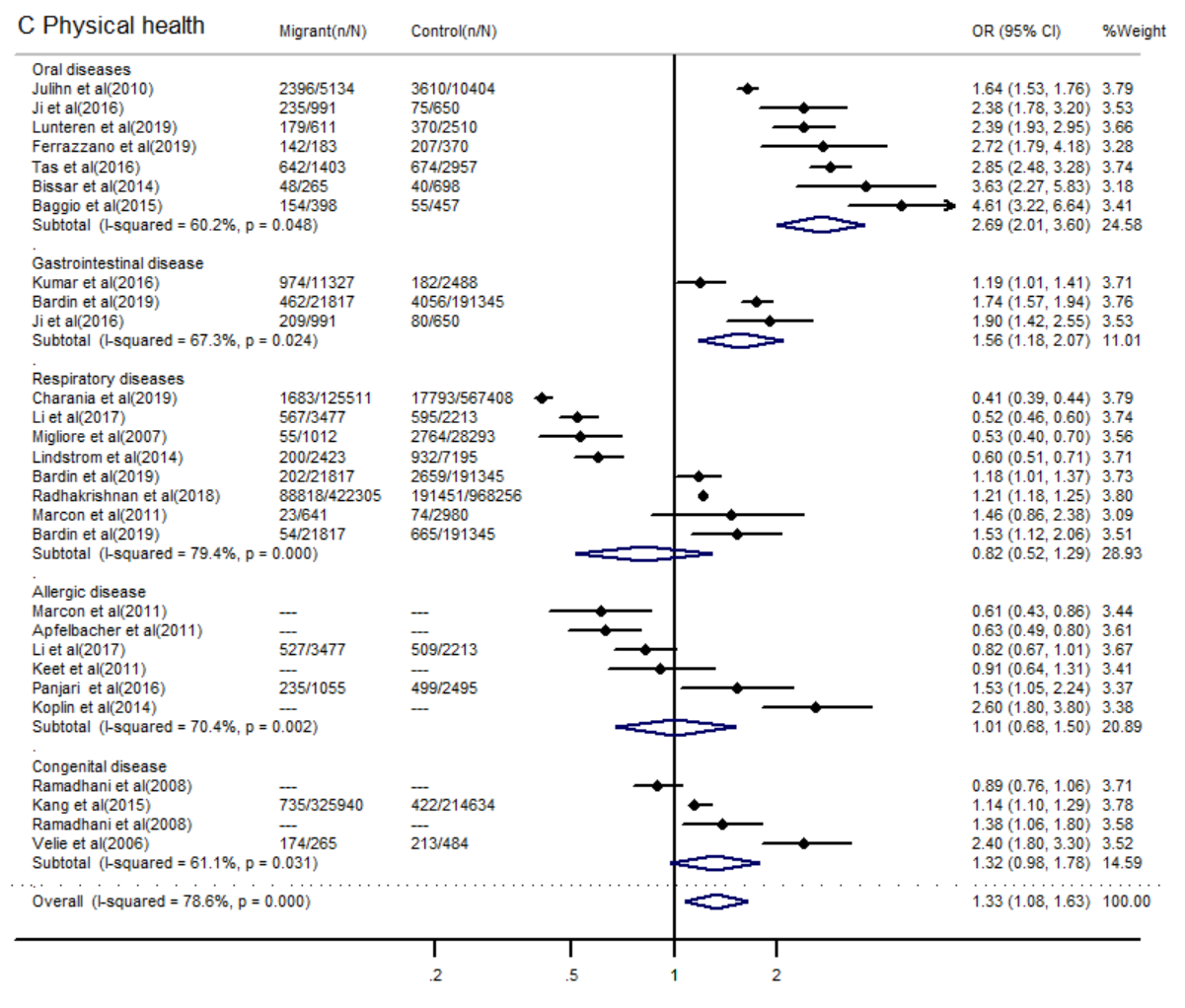


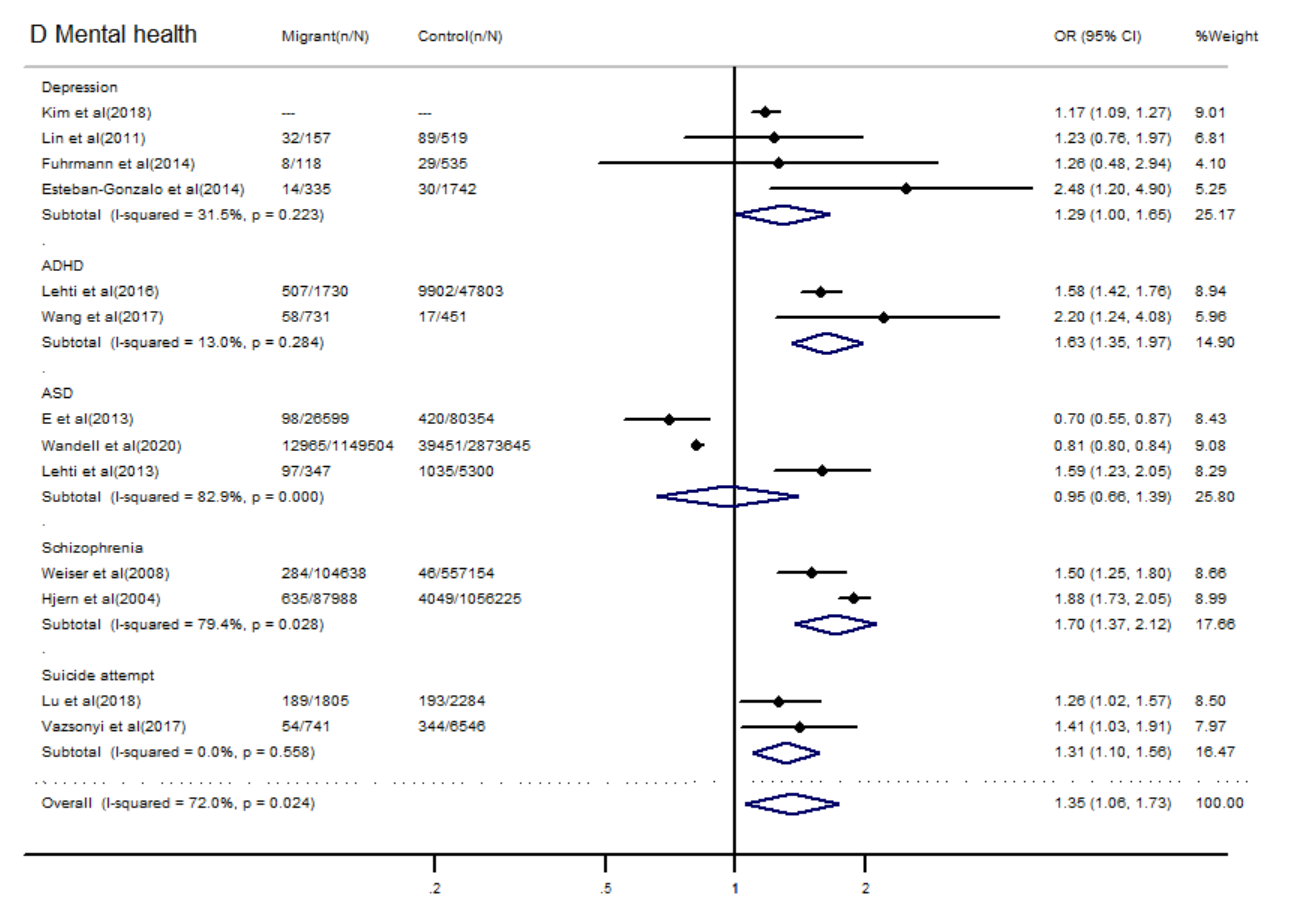


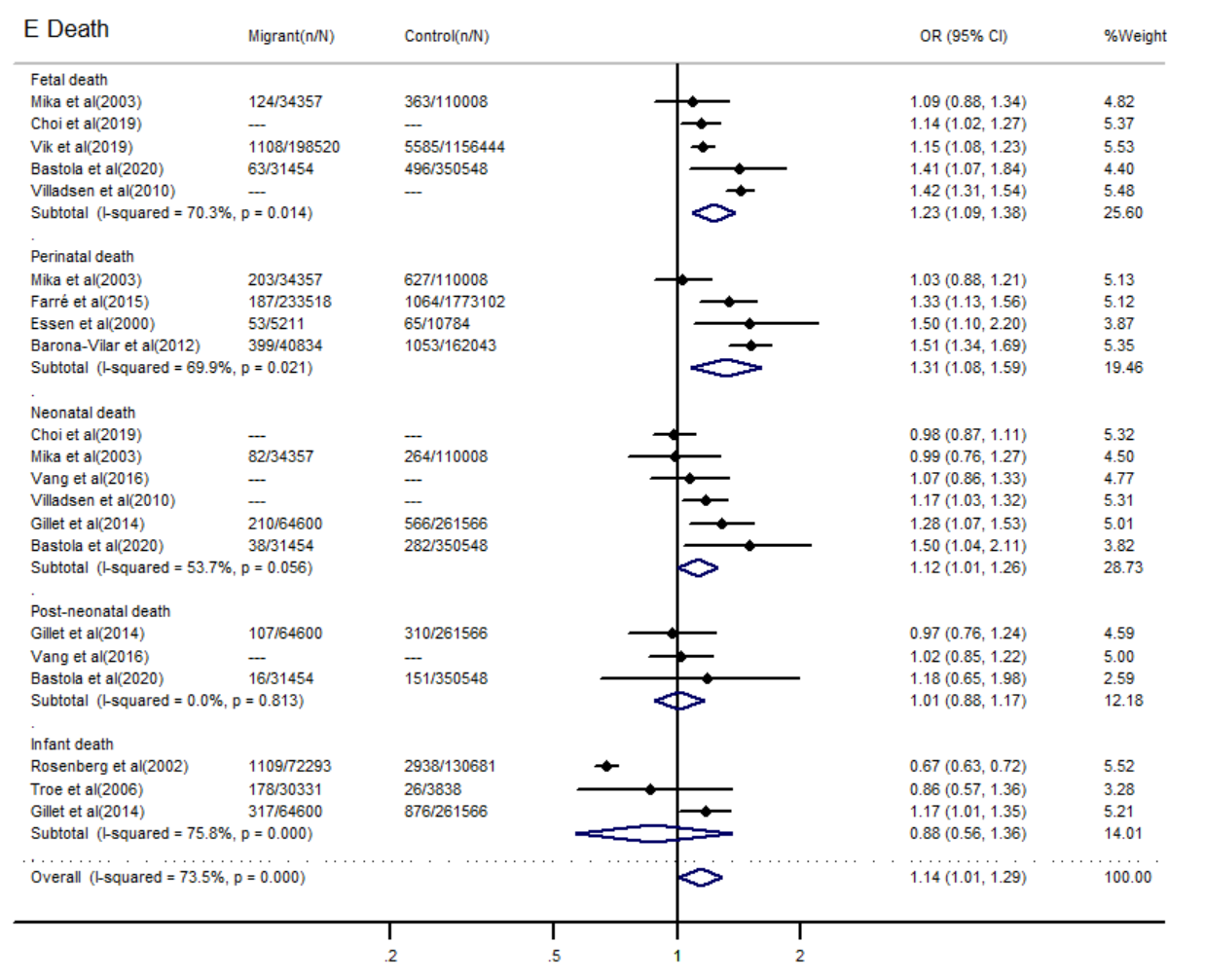


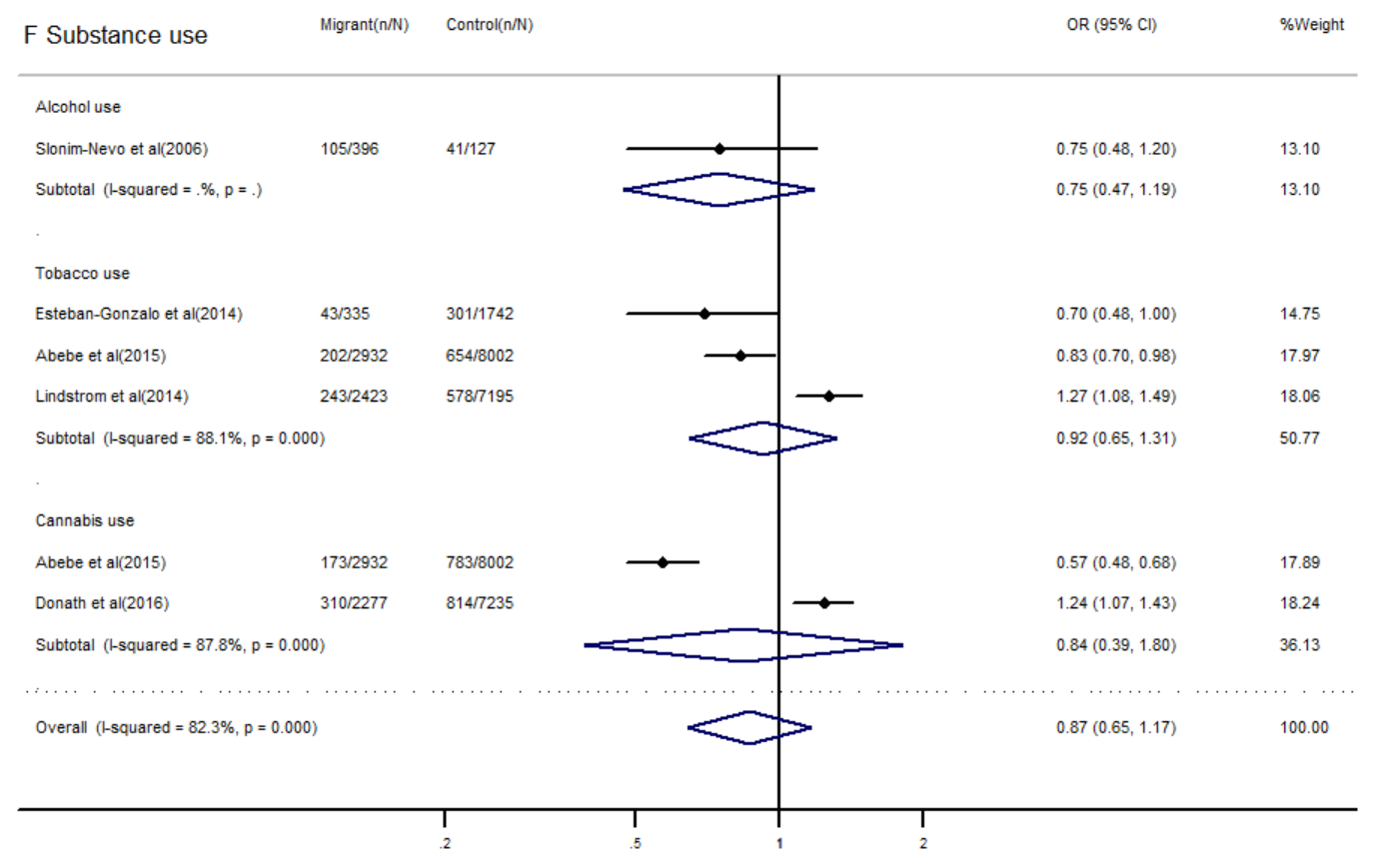


**Supplement figure1 Sensitivity analyses**
